# Pathogenesis, diagnosis and management of dentin hypersensitivity: an evidence-based overview for dental practitioners

**DOI:** 10.1186/s12903-020-01199-z

**Published:** 2020-08-06

**Authors:** Xiu-Xin Liu, Howard C. Tenenbaum, Rebecca S. Wilder, Ryan Quock, Edmond R. Hewlett, Yan-Fang Ren

**Affiliations:** 1 Department of General Dentistry, Eastman Institute for Oral Health, University of Rochester, 625 Elmwood Ave, Rochester, NY 14620 USA; 2 Department of Dentistry, Atlanta VA Medical Center, Atlanta, GA USA; 3 Department of Periodontology, Faculty of Dentistry, University of Toronto, Toronto, Ontario Canada; 4 Office for Professional Development and Faculty Affairs, Adams School of Dentistry, University of North Carolina-Chapel Hill, Chapel Hill, North Carolina USA; 5 Department of Restorative Dentistry & Prosthodontics, University of Texas School of Dentistry at Houston, Houston, TX USA; 6 Section of Restorative Dentistry, UCLA School of Dentistry, Los Angeles, California USA; 7 Outreach and Diversity, UCLA School of Dentistry, Los Angeles, California USA

**Keywords:** Dentin hypersensitivity, Hydrodynamic theory, Dental pain, Dental erosion, Adenosine triphosphate

## Abstract

Though dentin hypersensitivity (DHS) is one of the most common complaints from patients in dental clinics, there are no universally accepted guidelines for differential diagnosis as well as selection of reliable treatment modalities for this condition. The neurosensory mechanisms underlying DHS remain unclear, but fluid movements within exposed dentinal tubules, i.e., the hydrodynamic theory, has been a widely accepted explanation for DHS pain. As several dental conditions have symptoms that mimic DHS at different stages of their progression, diagnosis and treatment of DHS are often confusing, especially for inexperienced dental practitioners. In this paper we provide an up-to-date review on risk factors that play a role in the development and chronicity of DHS and summarize the current principles and strategies for differential diagnosis and management of DHS in dental practices. We will outline the etiology, predisposing factors and the underlying putative mechanisms of DHS, and provide principles and indications for its diagnosis and management. Though desensitization remains to be the first choice for DHS for many dental practitioners and most of desensitizing agents reduce the symptoms of DHS by occluding patent dentinal tubules, the long-term outcome of such treatment is uncertain. With improved understanding of the underlying nociceptive mechanisms of DHS, it is expected that promising novel therapies will emerge and provide more effective relief for patients with DHS.

## Background

Dentin hypersensitivity (DHS) is one of the most common complaints from patients in dental clinics. DHS has been defined as a short, sharp pain that arises from exposed dentin in response to non-noxious stimuli, typically thermal, evaporative, tactile, osmotic or chemical, and that cannot be ascribed to any other form of dental defects or diseases [1] . Studies have demonstrated vast variations in the prevalence of DHS, ranging from 1 to 98% [2]. The wide variability in prevalence likely results from variations in the criteria used to define DHS and from exclusive reliance on data derived from questionnaires as opposed to reliable clinically-based parameters in addition to patient histories [3–5].

In patients with DHS, the affected teeth become sensitive to generally non-harmful environmental stimuli. Gentle touch, mild cold or hot, chemical (acidic or sweet fruits, foods, drinks) and air-flow stimuli can induce short sharp pain that may affect daily activities including eating, drinking, speaking and tooth brushing. More severe DHS can last more than 6 months and become a consistent annoyance, inducing psychological and emotional distractions [6, 7], which may trigger the development of chronic dental pain condition requiring management as neuropathic pain. It is also known that the oral health related quality of life in patients with DHS can be improved after DHS has been treated successfully [8].

Even though DHS is one of the most common problems encountered by dental professionals, universally accepted guidelines for differential diagnosis as well as selection of reliable treatment modalities are lacking. While the neurosensory mechanisms underlying DHS remain unclear, the notion that DHS is related to fluid movements within exposed dentinal tubules, i.e., the hydrodynamic theory, remains a widely accepted explanation for DHS pain. Yet even this theory cannot account for all pain associated with DHS, nor does it necessarily lead to effective treatments that are consistent with this theory as the sole explanation for DHS. Problems associated with diagnosis and treatment of DHS are further exacerbated by the fact that several dental conditions have symptoms that can mimic DHS at different stages of their progression. The resultant diagnostic challenges for dental professionals are considerable, especially for novice practitioners or trainees, leading to delays in treatment and unnecessary suffering for patients. Based on a survey of dentists and dental hygienists conducted by the Canadian Advisory Board on Dentin Hypersensitivity, approximately 50% of respondents reported a lack of confidence in managing patients’ DHS-related pain [3].

In this paper we provide an up-to-date review on the putative etiological factors that play a role in the development and chronicity of DHS and summarize the current principles and strategies for differential diagnosis and management of DHS in dental practices.

### Etiology

Even though DHS has been recognized as a clinically important dental problem for more than a century, the exact pathogenesis, particularly in regard to the pain transduction mechanisms that play a role in DHS remain to be elucidated. Our understanding about its etiology is based primarily on data obtained from in vitro and in situ studies as well as from data obtained from epidemiological surveys [9]. It is generally regarded that DHS is associated with dentin exposure, especially exposure of open dentinal tubules, and dental pulp nerve responsiveness to external environmental stimuli [10].

Dentin exposure can be caused by physical, chemical, pathological, biological challenges and/or developmental abnormalities that result in dental and/or periodontal damage or defects. Various clinical conditions thought to play a role in the development of DHS include enamel attrition [11] and erosion [12], corrosion [13], abrasion and abfraction [14]. Periodontal tissue loss or gingival recession is another major predisposing factor since this leads to exposure of cervical and root dentin [9, 15]. Other factors, such as aging, soft tissue dehiscence, including aggressive brushing, can also cause apical displacement of the gingival margins thereby leading to exposure of dentin that can ultimately lead to the development of DHS [16].

### Symptoms and putative mechanisms of DHS

Initially, DHS presents as an acute pain that can be described as an unpleasant sensory experience in response to non-harmful or potentially harmful stimuli. A number of theories have been proposed to explain pulpal nociceptive transduction observed with DHS. One early hypothesis held that dentin was innervated and therefore nociceptive nerve endings within dentinal tubules were activated directly as stimulation was applied to the exposed dentin. The theory of direct dentin stimulation was abandoned because of the lack of evidence of dentin innervation based on various assessment including immunohistochemical and ultrastructural analyses [17]. Since odontoblasts are located at the outermost layer of the dental pulp and send processes into the dentinal tubules toward the dentinal enamel junction, it had been proposed that odontoblasts or at least their processes might themselves act as pain receptors, thereby transmitting pain signals to pulpal nerves that might be associated with the body of the odontoblasts within the pulp [18]. There is, however, no evidence confirming that synaptic structures that might link odontoblasts with pulpal nerves actually exist [19, 20].

The most widely accepted mechanism for DHS has been the hydrodynamic theory proposed by Brännström [21]. It states that environmental, mechanical, thermal, and chemical changes cause the movement of fluid within dentinal tubules, which stimulate the terminals of pulpal nerve fibers located within the tubule inlet walls, thereby inducing transient acute pain. The hydrodynamic theory highlights the concept that a number of different stimuli can evoke similar responses. Evaporative stimuli such as air blast as well as thermal (cold) and osmotic (sugar, acid) stimuli are thought to increase the outward flow of tubular fluid [22]. Mechanical stimuli such as a dental instrument or a toothbrush drawn across an exposed dentin surface are thought to compress the surface tissue, with the expansion upon release triggering an increase in outward flow of fluid [23]. The intra-dental myelinated Aβ and some Aσ fibers that send terminals into the dentin tubules are thought to respond to the fluid movements within the tubule resulting in the characteristic short, sharp pain of DHS [22]. However, how these essentially non-noxious mechanical stimuli of dentin tubule fluid movements induce the nociceptive transduction in dental pulpal nerve fibers remains an enigma. It has been shown that DHS could persist even when dentin tubules were deliberately obturated with gutta percha and fluid movements became impossible [24]. The clinical fact that the symptom of dentin sensitivity remains or exacerbates when the dentin tubules are obliterated by caries progression further undermines the notion that the hydrodynamic theory can explain all forms of DHS.

The hydrodynamic theory has indeed been challenged by emerging evidence suggesting that odontoblasts might well play an important role in pathogenesis mechanisms of DHS [25–27]. The odontoblastic processes in dentin tubules are the first dental pulp cells to detect external stimuli in the presence of dentin exposure. Though a physical synaptic structure is absent, dental pulp nerve fibers are in close proximity to odontoblasts and tightly entangle these cells. This finding could explain how signals could be transmitted to adjacent nerve endings through chemical mediators released from the odontoblasts despite the absence of physical synapses. That is, paracrine cell-cell communication plays a role in signal propagation as opposed to classic neural synapses. For the past decade, chemo-, mechano-, and/or thermo-sensitive channels such as connexin, pannexin, TRPV1, TRPV2, TRPV3, TRPV4 TRPM3, KCa, TREK-1, beta-ENa(+) C and ASIC2 channels have been identified in odontoblasts [25, 28–33]. These channels are also found in high levels in sensory neurons which could support the concept that odontoblasts might actually have the capacity to act as sensory cells to mediate or modulate pain transduction in dental pulp nerve fibers. Indeed, it has been demonstrated that mechanical and/or thermal stimulation induces the release of pain mediators such as adenosine triphosphate (ATP) and glutamate from odontoblasts [28, 29, 34], providing further evidence supporting the idea that these cells express, at least in part, a neurosensory cell phenotype. In addition, mechanically stimulated induction of ATP release and ATP-mediated signal transmission from odontoblasts to trigeminal neurons has been demonstrated in vitro using co-culture models comprised of odontoblasts and trigeminal neurons [31, 35]. Recently, the existence of autocrine/paracrine mechanisms for ATP-involved purinergic signaling in cultured odontoblast-like stem cells has been detected [36]. If this is the case, then the need for the presence of synaptic structures associated with odontoblasts for them to participate in the transmission of pain signals is obviated. Together, mounting evidence indicates that external stimuli-induced mechanosensitive responses from odontoblasts and subsequent nociceptive transduction in pulpal nerves may represent a novel explanation as to how odontoblasts participate in a mechanosensory mechanism leading to the pain associated with DHS [9] .

### Diagnosis of DHS

In order to determine an appropriate and effective treatment, it is critical to arrive at a correct diagnosis for DHS. A definitive diagnosis for DHS is usually reached through exclusion of other conditions that need varieties of treatment options. Any condition causing dentin exposure, dental pulp hyperemia, dental nerve sensitization and neuropathy may induce short sharp pain even with only minor provocation. A number of other conditions that give rise to similar symptoms of DHS need therefore to be distinguished [19]. Dental caries remains asymptomatic until the lesion reaches the dentin and affects the dental pulp, and then a series of conditions ranging from reversible pulpitis to irreversible pulpitis and apical periodontitis may develop in succession. DHS-like symptoms are often present at early stages of carious pulp involvement and it is essential to first rule out caries-related pain in the differential diagnosis of DHS [20, 37]. Other conditions that may present similar symptoms of DHS include cracked teeth, defective or fractured restorations, (usually recent) tooth preparation for restorations or restoration-induced pulp hyperemia, tooth whitening, dental trauma, occlusal trauma, cervical plaque and gingivitis, periodontal disease and its treatment, and other dental pulp/endodontic problems [2]. It is also necessary to point out that multiple conditions can co-exist in patients with DHS and thus exacerbate its symptoms. For example, peroxide agents used in tooth whitening can penetrate directly into the dental pulp thus inducing pain sensitization by affecting odontoblasts [38, 39], and this can aggravate DHS further. The proposed protocols for differential diagnosis of DHS include chief complaint and symptom inquiry, present illness history review, clinical exam and diagnostic testing. Regarding diagnostic testing, one of the most reliable outcomes would result from stimulating the involved tooth using a triggering stimulus reported by the patient, verifying that the patient’s chief pain complaint can actually be triggered.

#### Chief complaint and illness history

The chief complaint provides primary subjective information for the diagnosis of DHS, but it can be misleading for inexperienced practitioners. DHS is characterized with transient sharp pain following various stimuli, and it should disappear immediately after removal of the external stimulus. Non-transient, lingering dental pain is usually a sign of inflammation within the dental pulp and/or periodontal tissues, as well as possible infection that is associated with a pathological condition such as caries, traumatic tooth fracture, pulp exposure, pulp sclerosis, periodontal or apicoperiodontal diseases. DHS usually occurs as a minor annoyance to the patients but may progress gradually to sharp pain elicited by daily activities such as drinking, eating and tooth brushing. Therefore, the intensity and quality of pain in patients with DHS can vary depending on the severity of dentin exposure and status of both nociceptive peripheral and central sensitization, but its stimulation-dependent and transient properties should be the same. It is important to note that DHS is always provoked subsequent to the delivery of an external stimulus, and rarely if ever presents as pain that is either continuous or spontaneous. In fact, if the latter two symptoms are reported, diagnoses other than DHS should be considered within the differential diagnostic process.

The loss of enamel as well as the presence of dentin exposure, which result from bruxism, acidic diet habits, gastric reflux and bulimia, are considered as predisposing factors that can lead to DHS and should always be evaluated. One should also consider the presence of other predisposing factors such as gingival recession, gingivitis, periodontitis (and its treatment), and overzealous tooth brushing. Appropriate inquiries about the symptom or complaint of patients and reviewing the history of DHS and its predisposing factors will help the practitioner to reach the correct diagnosis and set the patients on the right track for treatment of DHS [40].

#### Clinical exam

Clinical examination and testing are requisite for differentiating DHS from other causes of hypersensitivity. Initially, the presence of exposed dentin must be identified by visual/tactile examination of the teeth and documented by site in the patient record. In addition, some indication of the exposure dimensions and/or photographs or casts are recommended to assess for exposure progression over time as an indicator of etiological factors (attrition, erosive or abrasive tooth wear activities). Gingival recession leading to exposure and wear of cementum is another predominant sign that should raise the level of suspicion for a diagnosis of DHS. Small erosive facets or shallow groves at the gingival margin are the most common findings in patients with DHS. However, dentin exposure may present but might not be readily identifiable by visual inspection, even in cases of DHS that is related to gingival recession depending on the extent of the recession. It is important to recognize that DHS most likely is only present at earlier stages of dentin exposure, that is, before the exposed dentin tubules become occluded and the dentin becomes sclerotic [41].

#### Diagnostic and differential diagnostic tests

In addition to visual inspection, it is essential to evoke or induce the characteristic transient sharp pain by applying a stimulus to the affected tooth to mimic the patient’s complaint or symptom. DHS can almost always be induced with an appropriate stimulus, particularly one that is related to the stimuli reported by the patient to trigger the DHS symptoms. No matter if dentin exposure is visually detectable or not, a gentle mechanical stimulus with a dental explorer drawn along the tooth (especially in the area indicated by the patient to be sensitive) can often elicit the transient pain and confirm the diagnosis. A stimulus that evokes DHS is often mild and would ordinarily not be expected to cause pain (somewhat similar to the description of allodynia) in healthy teeth. Depending on the patient’s complaint, mechanical, tactile and thermal stimuli or air blast with an air-jet, can be applied to the location of suspected dentin exposure. It is noteworthy that when blowing air or delivering other stimuli to an exposed root surface where there is minimal gingival recession or root exposure, care should be taken to cover the soft tissues or to carefully note that the soft tissues are not being stimulated along with the exposed dentin. It is possible in some cases that blowing air or stimulating the nearby soft tissues mechanically may actually activate a neuralgic trigger point (e.g. in trigeminal neuralgia or other neuropathic conditions) [42]. But in the absence of other diseases or pathological condition, the diagnosis of DHS can be confirmed if the outcome of the test is a transient sharp pain that matches the patient’s complaint. Based on the pain response, cold and hot stimulation can also be applied in order to distinguish a diagnosis of DHS from reversible and irreversible pulpitis (these latter two conditions would also generally also exhibit lingering pain). DHS is usually not induced by occlusal forces or to percussion unless the site of dentin exposure is on the occlusal surface. However, localized DHS-like pain that is evoked by occlusal force or by percussion could suggest other diagnoses including occlusal trauma, dental trauma, periodontal and apical periodontal problems or a cracked tooth (itself possibly caused by occlusal trauma).

As noted above, a cracked tooth can, when stimulated in certain ways, produce pain that is somewhat similar to that of DHS. Pain from a cracked tooth or a cracked/defective or poorly made restoration can be distinguished from DHS by instructing a patient to bite and then ‘roll’ their bite over a cotton swab. A diagnostic instrument, the Tooth Slooth® can also be utilized for this purpose with excellent results in helping to identify or rule out a cuspal fracture. In addition, transillumination and optical magnification can aid significantly in visualizing cracks. Radiographic examination is of limited value in confirming a diagnosis of DHS. Nevertheless, radiographic assessment is still required in order to rule out other pathological conditions such as caries, overt tooth fractures, defective restorations, or other dentoalveolar lesions that might cause pain.

**Fig. 1:**
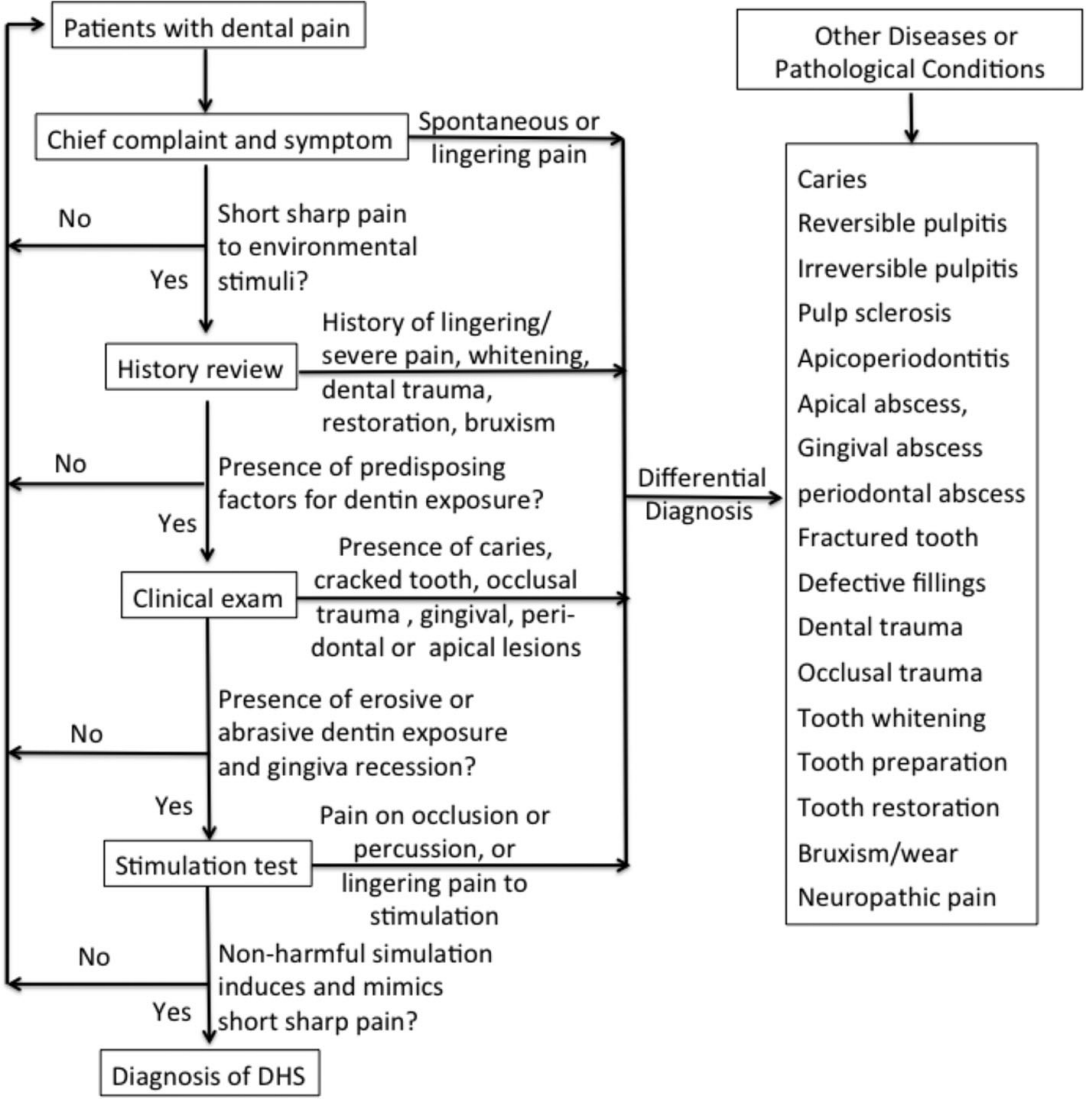
Flow chart for differential diagnosis of dentin hypersensitivity (DHS). The diagnostic process for DHS includes reviewing chief compliant, illness history and predisposing factors, and clinical exam, stimulation test, and radiographic examinations, if necessary

In light of the foregoing discussion then it would be appropriate to conclude that DHS can be diagnosed directly but is also a diagnosis of exclusion. This process is illustrated in Fig. 1 and further explained in Table 1.

**Table 1:** Diseases or Conditions to be excluded for the diagnosis of dentin hypersensitivity

Dental caries:	Severe sensitivity experienced when dental caries passes the dentin-enamel junction and affects the pulp
Cracked tooth syndrome	Sharp intermittent pain elicited on biting as the occlusal force increases, and relief of pain occurs once the pressure is withdrawn using bite test, a Tooth Slooth, or tapping of an individual cusp
Traumatized or chipped tooth	a. Enamel fracture: with superficial, rough edges that may cause tongue or lip irritation, but no sensitivity or pain b. Enamel and dentin fracture: with rough edges that usually accompanied with tooth sensitivity or pain
Pulpitis	a. Reversible pulpitis: with sharp pain that is provoked by hot, cold, or sweet stimulus. The pain last less than 20s after stimuli withdrawal b. Irreversible pulpitis: with severe, sharp, throbbing, intermittent or continuous pain that may keep the patient awake at night. Pain is provoked by cold, hot, chewing, lying flat and persists after stimuli withdrawal, and pain irradiating from other sites in the mouth (referred pain).
Periodontal abscess	Continuous dull pain that is aggravated on biting, often associated with deep periodontal pockets and alveolar bone loss
Periapical periodontitis	Continuous dull pain that is aggravated on biting, often associated with deep caries and a necrotic pulp
Pericoronitis	Continuous dull pain that is aggravated on biting, often associated with swollen pericoronal tissues
Bleaching sensitivity:	Pain resembles that of reversible pulpitis due to penetration of the bleaching agent into pulp chamber.
Tooth grinding (bruxism)	Pain and sensitivity to cold and hot stimuli due to occlusal wear, with dentin exposure associated with reflexive and repetitive chewing actions. May be accompanied by facial pain, tension headaches, stiffness and pain in the temporomandibular joint. Enamel micro-fractures and broken or chipped tooth may also occur.
Post-operative sensitivity	**a. Cavity preparation phase:** • Heat generation due to inadequate cooling during cutting • Exerting excessive pressure during cutting • Vibration due to eccentricity of the bur • Dentin desiccation which contributes to sensitivity of vital dentin to any subsequent irritant. **b. Restorative phase:** • For composite resin restoration, post-restorative hypersensitivity may be related to leakage, improper bonding procedure, cuspal strain, or a fractured restoration • For amalgam restoration, post-restorative hypersensitivity may be associated with lack of effective dentin insulation, leakage, hairline cracks, fractured restorations, premature contacts or galvanic stimuli. • Post-operative sensitivity associated with resin cements used for cementation of indirect restorations

### Management of DHS

**Fig. 2:**
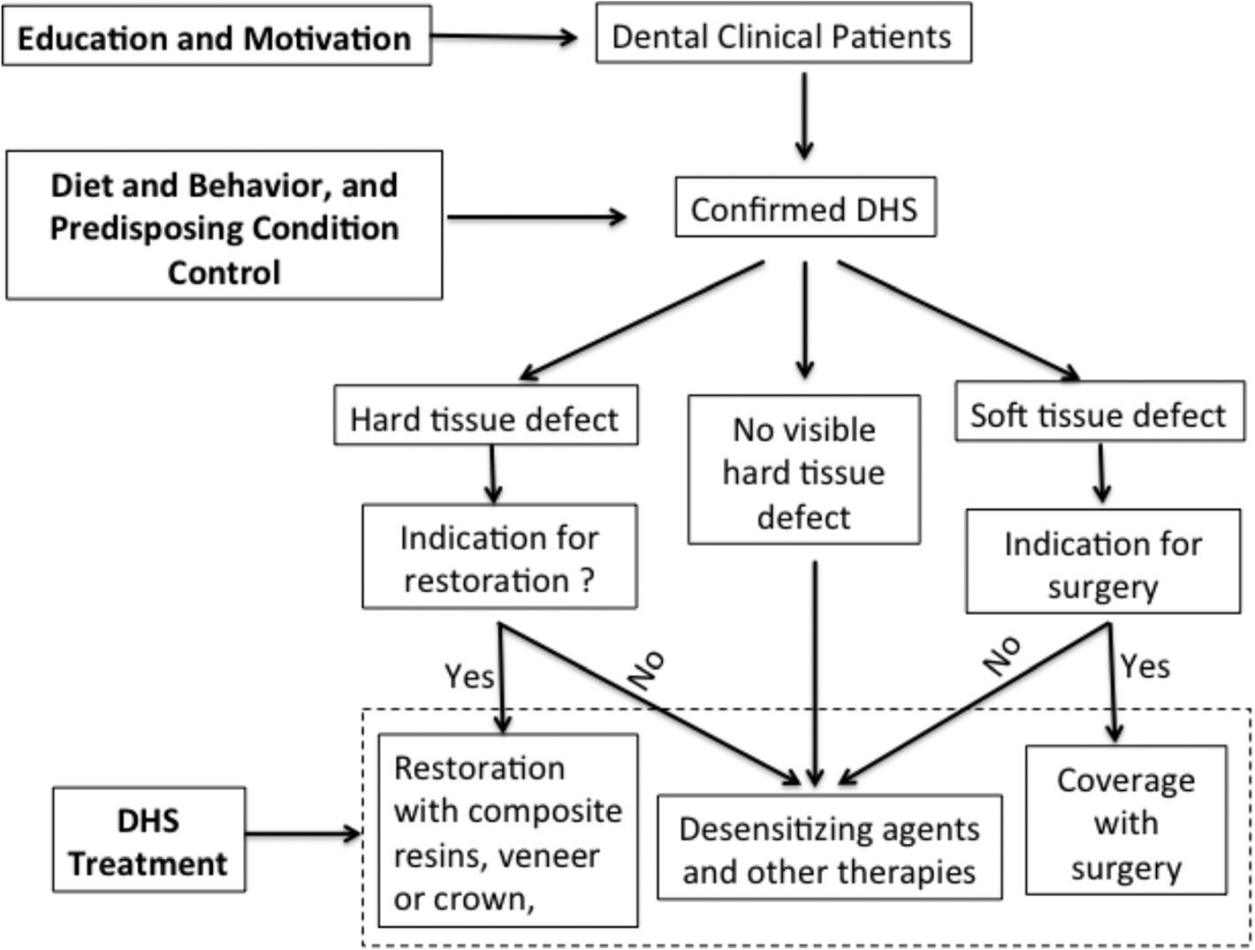
Strategies for managements of dentin hypersensitivity (DHS)

Strategies for management of DHS include: 1) Oral hygiene education and brushing technique instruction for prevention of DHS; 2) Behavioral control and elimination of predisposing factors for DHS; 3) Non-invasive treatments for pain relief through occluding dentin tubules and blocking nociceptive transduction/transmission. 4) Restoration or surgical treatments for dental hard and soft tissue defects. A flow chart of the decision-making process for DHS management is shown in Fig. 2.

#### Oral hygiene instruction for DHS prevention

Given that DHS is largely the result of erosive/abrasive tooth wear or gingival recession related dentin exposure, dental practitioners should employ preventive strategies directed at known predisposing etiological factors for DHS [40, 43]. Education, instruction, and engagement in prevention of erosive and abrasive tooth wear and gingival recession should be routinely provided to dental patients. Since the acids from vinegar, fruit and fruit juices, as well as soft drinks (e.g. citric, malic, and phosphoric acid) are the major cause for dental erosion, consumption of the acidic food or beverages should be regulated in patients prone to the development of DHS [44, 45]. Patients should also be made aware that other foods or beverages, although non-acidic, can contribute to lower pH in the oral cavity. These may contain various sugars or starches, which when broken down to their constituent sugars by salivary amylases, can lead to bacterial production of acid (lactic for example). Consuming such foods or beverages *before* removal of the oral biofilm can thus elevate susceptibility of exposed dentin surfaces to mechanical abrasion, even from gentle tooth brushing [46]. Hence, patients should be educated to brush their teeth prior to consumption of these foods and beverages [47]. Tooth brushing techniques such as selection of soft bristle brush and non-abrasive toothpaste, and using vertical sweeping motion that minimize injury to dental soft and hard tissues should be emphasized [20, 48]. However, if the dentinal surface has been ‘softened’ by biofilm-mediate acid production, the use of the softest toothbrushes, even without dentifrice can still cause wear of dentin [46].

#### Behavioral control and elimination of predisposing factors for DHS

In order to achieve long-term effective treatment or prevent further or new development of DHS, it is essential to eliminate predisposing factors causing dentin exposure. This includes control of acidic food or beverage consumption and eating habits as mentioned above. In cases with tooth wear caused by bruxism or compromised dentition, it is recommended that the use of an occlusal guard or restoration of the worn dentition and vertical dimension be done. As stated previously, gingivitis, periodontitis and their treatment have been identified as predisposing factors for DHS due to the secondary dentinal exposure that may result. This should be anticipated during periodontal treatment, and appropriate measures should be taken (e.g. modulation of other risk factors noted above) prior to, during and after treatment of gingival diseases for successful management of DHS [19] [40].

Excessive frequency of brushing in the absence of acid-mediated softening of dentinal surfaces has also been noted in many subjects plagued by DHS [46]. Overzealous brushing and other mechanical causes of gingival recession, e.g., the presence of tongue rings and studs should thus be considered for elimination or removal.

Medical and psychiatric conditions may contribute to dental erosion/abrasion and gingiva recession. Gastric reflux, the release into and the retention of gastric acids within the oral cavity, can erode both enamel and dentin aggressively leading to softening of surface dentin, thereby predisposing it to accelerated wear. Esophageal constriction/atresia due to injury (e.g. chemical) or disease (e.g. scleroderma) can also lead to increased levels of gastric acids in the mouth. Similarly, psychiatric disorders associated with binge/purge behavior (e.g., bulimia nervosa) subject teeth to destructive levels of gastric acid exposure. In any case, medical and/or psychiatric causes of DHS must be identified and be treated or controlled [49].

#### Non-invasive treatments for pain relief

Application of desensitizing agents is the most frequently used non-invasive treatment for DHS. Especially in cases with limited or invisible dental hard tissue loss or cervical exposure (i.e. no obvious erosive defects, classical abrasive lesions or gingival recession), the use of desensitizing agents or other analgesic treatments should be considered. Conceptually, desensitizing agents or analgesic treatments aim to suppress nerve impulses by either mechanical or chemical blockage of the dentin tubules or by directly stopping the nociceptive transduction/ transmission occurred within dentin-odontoblasts-nerve terminal complex of the dental pulp. Based on the mode of their administration, the desensitizing treatment can also be classified into at-home therapy or in-office therapy categories. At home desensitizing products include toothpastes, mouthwashes and chewing gums. In-office desensitizing products can be found in the form of gels, solutions, varnishes, resin sealers, glass ionomers, and dentin adhesives. In-office desensitizing treatments also include more sophisticated laser techniques. In general, all interventions should start with noninvasive, reversible, nonhazardous, easy to perform, and inexpensive options [50].

But how do these products work? It would appear that the active compounds found in desensitization products could either block the openings of dentinal tubules thereby isolating the tubule contents or might directly desensitize the pulpal nerves. Potassium salts were thought to decrease the excitability of pulpal nerves and result in a reduction in dentin sensitivity [51, 52], but clinical trials with sound design have failed to provide evidence that potassium is effective in desensitizing teeth [53]. It is likely that potassium salts reduced the perception of dentin sensitivity through a placebo effect [52]. The proposed mechanism for glutaraldehyde, another agent used for treatment of DHS, involves the reaction with serum albumin in dentinal tubule fluid, leading to precipitate formation within tubules, and subsequent narrowing or blocking of the tubules [54, 55]. Strontium salts [56], fluoride [57], oxalate [58] and arginine/calcium [59, 60] containing products have been demonstrated to precipitate and occlude the tubules and form a protective layer at the dentin surface. Dental adhesives and resin sealants can be used to seal the dentin tubules by forming a physical barrier thereby blocking dentinal fluid flow while also preventing direct stimulation of odontoblastic processes within dentinal tubules by outside stimuli [61]. The action of glass ionomers in management of DHS can also lead to occlusion of open dentinal tubules by precipitating a hydroxycarbonate apatite layer over the previously patent tubule openings.

Laser treatment has been investigated as a prospective treatment for DHS. Both low-output and higher output laser application have been reported to be effective for treatment of DHS. The mechanisms involved in laser treatment of DHS are not understood fully at this time though and still need to be elucidated [62]. Some experiments suggest that low output laser might operate by suppressing the excitability of pulpal nerves [63]. Higher output laser is thought to reduce symptoms of DHS by inducing the occlusion of dentin tubules [64]. Even though high output laser may not lead to adverse effects (provided that adequate care is used) when treating DHS, more consistent studies should be conducted in order to demonstrate more clearly whether or not these therapeutic approaches actually provide benefit to patients with DHS [63].

There is a plethora of agents, materials, and products for desensitization treatment of DHS available on the market. Recent systemic reviews and meta-analysis showed that most chemical or physical occlusion of dentin tubules agents had a statistically significant difference from placebo in reducing the symptom of DHS [65, 66]. Due to the environment of the oral cavity and the persistent existence of predisposing factors for DHS, retention of these occluding agents inside the patent dentine tubules is a challenge, and their long-term effects for the treatment of DHS remains unclear.

#### Restorative treatments or correction of hard and soft tissue defects in DHS

Direct restoration of hard tissue defects or surgical correction for gingival recession should provide an alternative treatment for DHS as indications for restorations or surgeries exist. For erosion or abrasion related DHS, it is believed that direct restoration with resin based composite or glass ionomer and indirect restoration with a crown or a veneer should provide effective long-lasting treatment for DHS [40, 61, 67]. Indications for direct hard tissue defect restorations are present in Table 2. Periodontal surgical procedures including guided tissue regeneration, coronally advanced flap surgery, connective tissue grafting, and free gingival graft treatments have been proposed for the treatment of DHS related to gingival recession, even though the long-term effects of these interventions are still being debated [68] (Table 3). Yet, the coverage of exposed dentin using mucogingival surgical methods does make sense from a practical perspective. However, if surgical correction cannot be attained, or even if there is some improvement in a recession defect and there are still symptoms of DHS, then other occlusive restorative treatments as outlined above need to be considered [69].

**Table 2:** Indications and limitations for non-invasive desensitization treatment for DHS

Indications for desensitization treatment for DHS	
○ No visually detectable defects of dental hard tissue	
○ Shallow or minimum hard tissue defect, where normal dental tissue has to be removed if restoration is provided.	
○ Hard tissue defect is esthetically acceptable and no predisposing factors exist for continued loss of dental tissues.	
○ Minimum gingiva recession and no predisposing factors exist for continued loss of gingiva and dental tissues.	
○ No other contraindications for desensitization treatment	
Risks and limitations for desensitization treatment	
○ Existence of predisposing factors for continued hard tissue lose	
○ Existence of predisposing factors for continued gingiva recession	
○ Existence of microfacture, caries and other dental problems present with similar symptoms of DHS

**Table 3:** Indications and limitations for restorations and mucogingival surgeries for DHS treatment

Indications for replacement of lost dentinal volume with restorations:	
○ Hard tissue defect is visible and requires restoration for esthetics and structural integrity	
○ Failure to resolve DHS using non-restorative treatment	
○ High risk for continued dental tissue loss if not covered with restoration (e.g. inability to halt erosive or abrasive tooth wear)	
○ Little or no removal of tooth structure is required for restoration	
Indications for mucogingival surgery	
○ Failure to resolve DHS using densensitization in patients with gingival recession	
○ Gingival recession is esthetically unacceptable to patient	
Risks or limitations for dental restoration and mucogingival surgery	
○ General impermanence of restorations - the re-restoration cycle	
○ Specific impermanence of resin-dentin bonding	
○ Increased risk of caries lesion at the site of restoration	
○ Questionable long term outcome of mucogingival surgery	

## Conclusion

The presence of dentin exposure is usually prerequisite to the development of DHS characterized by short sharp pain in response to various external, and usually non-noxious stimuli. In this review we have outlined the etiology, predisposing factors and the underlying putative mechanisms of DHS, and provided principles and indications for its diagnosis and management. Accurate diagnosis is the key in selecting the right treatment strategy for DHS (or any other condition for that matter). Desensitization remains to be the first choice for DHS and most of desensitizing agents reduce the symptoms of DHS by occluding patent dentinal tubules, but the long-term outcome of such treatment is unclear. Based on thorough reviews of the putative etiological factors in the development of DHS, we developed schemes for differential diagnosis and management decision-making that could be used as practical guidelines for diagnosis and treatment of DHS in general dental practices. These schemes should especially be helpful to dental students or practitioners who are not experienced in DHS diagnosis and management. We anticipate that with improved understanding of the underlying nociceptive mechanisms of DHS, promising novel therapies will emerge and provide more effective relief for patients with DHS.

## Data Availability

Not applicable.

## Abbrevations

DHS: dentin hypersensitivity.
ATP: adenosine triphosphate.
